# Chirp-modulated light-induced thermoelastic spectroscopy for simultaneous precise measurement of resonant frequency and gas concentration

**DOI:** 10.1016/j.pacs.2026.100812

**Published:** 2026-02-16

**Authors:** Xiang Chen, Xinyao Chen, Lu Yao, Mai Hu, Hao Liu, Ruifeng Kan

**Affiliations:** a Jinling Institute of Technology, Nanjing 211169, China; b Hefei Institute of Physical Science, Chinese Academy of Sciences, Hefei 230031, China

**Keywords:** Absorption spectroscopy, Light-induced thermoelastic spectroscopy, Quartz tuning fork, Chirp modulation, Resonant frequency, Gas concentration

## Abstract

This paper demonstrates chirp-modulated light-induced thermoelastic spectroscopy (LITES), enabling simultaneous precise measurement of quartz tuning fork (QTF) resonant frequency and gas concentration. The injection current of the laser is modulated using the chirped signal. The chirped laser beam is injected into a multi-pass cell and the output beam is then focused onto the root of the QTF. Adopting the chirped signal as the reference signal, a lock-in amplifier is used to obtain the first harmonic (1 *f*) signal. The 1 *f* signal can be well-fitted with the Lorentz profile. Based on the peak position and area of the 1 *f* signal, the QTF resonant frequency and gas concentration can be obtained respectively. Compared with the traditional electric excitation method, the deviation of the calculated resonant frequency is only 0.02 Hz. This technique is able to prevent QTF frequency drift from affecting precision measurements, thereby guaranteeing the long-term stability of the LITES system.

## 1 Introduction

Laser absorption spectroscopy has become a core analytical technology in trace gas analysis due to its advantages of strong anti-interference capability, high sensitivity, and fast response [1], [2], [3], [4], [5], [6]. This technology shows broad application prospects in scenarios such as environmental pollution monitoring [7], [8], [9], marine dissolved gas detection [10], [11], medical diagnostics [12], [13], and industrial process control [14]. Among them, quartz-enhanced photoacoustic spectroscopy (QEPAS) is a technique that measures gas concentration by detecting the acoustic signal generated during the absorption process of gas molecules using a QTF [15], [16], [17]. The QTF must be placed within the target gas, and exposure to corrosive gases can degrade its performance. In contrast, the LITES employs the QTF to measure transmitted light intensity after gas absorption [18], [19], [20], [21], without requiring direct contact with the gas molecules, thereby ensuring stable performance of the QTF. In LITES, the QTF converts absorbed thermal energy into mechanical energy, exciting the QTF arm to vibrate and generate piezoelectric signals, thereby enabling the detection of spectral signals [22], [23], [24]. Compared with conventional photodetectors [25], [26], [27], QTF provides advantages such as extremely wide spectral response (0.2–10 μm), exceptionally high quality factor (>10⁵), and low manufacturing cost [28], [29], [30].

The frequency response curve of the QTF can be described by the Lorentz profile, whose characteristics are highly sensitive to external environmental conditions [31], [32], [33], [34]. The thermal expansion coefficient of the QTF substrate material causes the QTF resonant frequency to exhibit a linear decrease with increasing temperature [35]. Reducing the environmental gas pressure or the molecular weight of the background gas can diminish the damping effect, resulting in increased quality factor and resonant frequency [36]. Moreover, prolonged exposure to light causes the temperature at the beam convergence point of the QTF to be higher than other parts, which can cause microscopic deformation and stress accumulation within the QTF, potentially triggering irreversible changes in material quality distribution and subsequently leading to resonant frequency shifts. However, the QTF has a narrow frequency response bandwidth, and the small frequency drift can significantly impact the spectral response, which will seriously compromise the long-term stability of the LITES system [37], [38], [39]. For commercial QTFs, a frequency drift of 0.5 Hz results in a reduction of approximately 10 % in spectral response. Moreover, frequency drift has a more severe impact on spectral response for narrow-bandwidth customized QTFs.

Currently, the majority of relevant studies employ the electric excitation method to measure the resonant frequency of QTFs [40], [41], [42], [43], [44]. Sinusoidal signals of different frequencies are generated and utilized to excite the QTF. The amplitude of the output signal at each frequency is measured and processed to obtain the precise resonant frequency [45], [46]. When changing between two adjacent modulated frequencies, sufficient response time is required to allow the QTF to reach a stable vibrational state at that frequency. This process typically takes several seconds. To obtain a more precise QTF resonant frequency, it is necessary to measure the QTF response at as many frequencies as possible. Hence, this method offers high measurement accuracy but requires a relatively long measurement time of several minutes. It also cannot achieve simultaneous measurement of resonant frequency and gas concentration, rendering it inadequate for real-time frequency compensation in applications. Beat-frequency quartz-enhanced photoacoustic spectroscopy enables simultaneous measurement of trace gas concentration and frequency parameters of QTF in either a differential-frequency modulation manner [47] or a differential-frequency demodulation manner [48]. For beat-frequency quartz-enhanced photoacoustic spectroscopy, the laser modulation frequency has a frequency difference with respect to the QTF resonant frequency. The harmonic signal is demodulated at the laser modulation frequency. By measuring the attenuation coefficient of this signal, information such as gas concentration and resonant frequency can be obtained. Ma et al. implemented a similar measurement technique in the LITES system to realize sensitive hydrogen detection [49]. Nevertheless, the measurement accuracy of the resonant frequency using this method is only in the Hz range. The conventional point-by-point frequency sweep method can theoretically be used for simultaneous measurement of the resonant frequency and gas concentration. However, during the process of changing the modulation frequency, due to the high Q-factor of the QTF, establishing or eliminating a particular vibration mode requires a certain amount of time for energy accumulation [50], [51]. Therefore, when changing between two consecutive modulation frequencies, sufficient response time is required to allow the QTF to reach a stable vibrational state at that frequency. For common commercial QTFs, this interval is approximately 8 s [52]. Therefore, this method requires about 8 s to measure the signal at a single frequency. To achieve a complete spectral measurement, it is typically necessary to measure spectral signals at dozens of frequencies, which can result in system response times lasting several minutes. In field measurements, gas concentrations are difficult to maintain constant for minutes. Variations in gas concentration can distort the measured spectrum, thereby significantly compromising detection sensitivity.

This paper investigates the chirp-modulated LITES system to address the requirements for high-precision simultaneous measurements of QTF resonant frequency and gas concentration. Theoretical calculations are performed primarily for the 1 *f* signal of the chirp-modulated LITES system, indicating the simultaneous measurement approach for QTF resonant frequency and gas concentration. Then, the correspondence between the frequency range of the chirped signal and the 1 *f* signal is measured, and the measurement accuracy of the resonant frequency is demonstrated. The variation trends of the 1 *f* signal under different modulation depths and durations are also investigated. To evaluate the system's response at different gas concentrations, calibration experiments are conducted across a wide concentration range. Finally, the minimum detection limit of the chirp-modulated LITES system is determined based on Allan variance analysis.

## 2 Principles of the 1 *f* signal of chirp-modulated light-induced thermoelastic spectroscopy

A chirped signal is employed to modulate the laser injection current. The chirped signal is a cosine signal with a linearly changing frequency, which can be expressed as:

(1) $$I_{chirp}=a\;\cos(2\pi (bt+f_{0})t+\varphi )+c$$

where *a* is the modulation amplitude, *b* is the frequency change rate, *f*_*0*_ is the initial frequency, *φ* is the initial phase, and *c* is the bias. The frequency response curve of a QTF can be described by a Lorentz profile [53]:

(2) $$S_{QTF}=\frac{Aw_{1}}{\sqrt{{\left(1-f_{c}/f\right)}^{2}+{w_{1}}^{2}}}+B$$

where *f*_*c*_ is the QTF resonant frequency, *w*_1_ is the frequency response bandwidth, and *B* is the bias. The integral of the Lorentz function over the entire frequency range is a finite value, which can be obtained through fitting. And *A* is the area of the frequency response curve. When a chirped laser beam is incident on the surface of a QTF, it excites the light-induced thermoelastic signal at the corresponding frequency. During the measurement process, the average output intensity of the laser remains constant. As the modulation frequency gradually varies and covers the resonant frequency of the QTF, the amplitude of the stimulated LITES signal aligns with the frequency response curve of the QTF. Consequently, the Lorentz function can be employed to characterize this signal. The signal is amplified using a transimpedance amplifier, and the amplified signal is then fed into a lock-in amplifier. Another chirp signal is employed as the reference signal, whose characteristic parameters are consistent with those of the chirped signal employed to modulate the laser injection current. The output signal of the lock-in amplifier is the 1 *f* signal. The corresponding 1 *f* signal can be expressed as:

(3) $$S_{1f}=k(\frac{Aw_{2}}{\sqrt{{\left(1-f_{c}/(bt+f_{0})\right)}^{2}+{w_{2}}^{2}}}+C)$$

In which *k* is the absorption coefficient, *w*_2_ is the 1 *f* signal bandwidth, *C* is the bias. It can be observed that the 1 *f* signal also conforms to the distribution of Lorentz profile. Hence, the QTF resonant frequency can be calculated based on the peak position of the 1 *f* signal. If target gas molecules are within the optical path, the absorption coefficient will decrease. The decrement is proportional to the gas concentration. Based on the above analysis, the chirp-modulated LITES system can realize simultaneous measurement of both the QTF resonant frequency and gas concentration.

## 3 Experimental setup

**Fig. 1:**
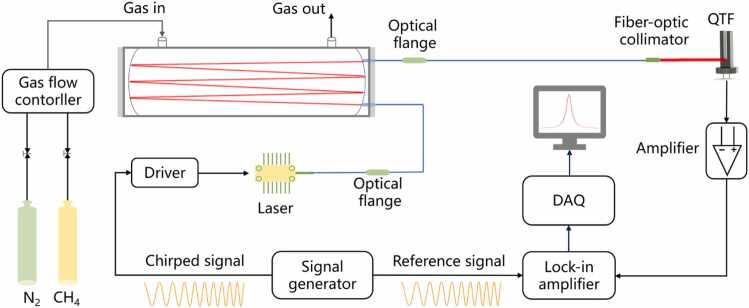
Experimental setup of the chirp-modulated LITES system.

Fig. 1 illustrates the chirp-modulated LITES system. A homemade waveform generator generates two chirped signals with identical parameters. The frequency scanning range, amplitude and period of the chirped signals can be freely adjusted. One of the chirped signals is adopted to modulate the injection current of a near-infrared tunable diode laser. To suppress the wavelength drift, we simultaneously and precisely control both the chip temperature and the shell temperature of the laser. The chirp-modulated beam is collimated and passes through a multi-pass cell with an optical length of 3 m. It then converges at the root of a commercial QTF to excite light-induced thermoelastic signals. The wavelength tuning coefficient of the laser is 0.01 nm/mA. By carefully adjusting the bias of the chirped signal, the laser's center wavelength is positioned at 1653.7 nm, and methane (CH_4_) is selected as the target gas. Different concentrations of standard gases are obtained with the Gas flow controller (Iknow-meter, IKFD-MM). The stimulated piezoelectric signal is amplified by a transimpedance amplifier and then is fed into a lock-in amplifier (Stanford, SR865A). The time constant of the lock-in amplifier is set to 100 ms. Employing another chirped signal as the reference signal, the 1 *f* signal can be demodulated. The 1 *f* signal indicates the frequency response of the QTF across the frequency range of the chirped signal. This method yields curves consistent with those obtained by directly calculating the frequency response of a QTF. Since the 1 *f* signal is commonly used in LITES systems, we employ the 1 *f* signal for subsequent experimental research. A data acquisition card (NI, USB-6363) is utilized to acquire 1 *f* signals with a sampling rate of 2 kS/s and a vertical resolution of 16 bits.

## 4 Experimental results

### 4.1 1 *f* signals at different frequency ranges of chirped signals

**Fig. 2:**
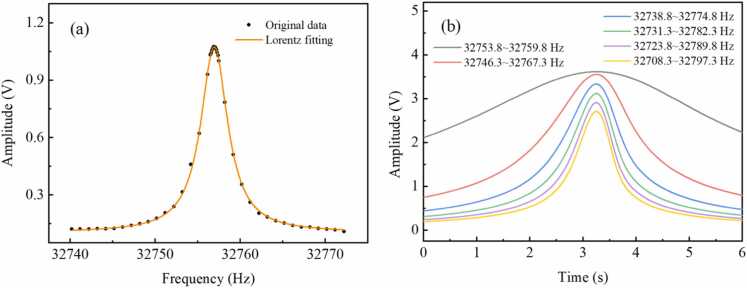
(a) QTF frequency calibration using the traditional electric excitation method, (b) 1 *f* signals at different frequency ranges.

**Fig. 3:**
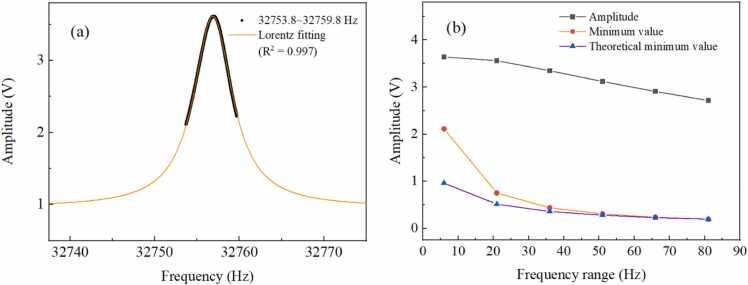
(a) Lorentz fitting of the 1 *f* signal, (b) characteristics of 1 *f* signals at different frequency ranges.

A commercial QTF is adopted in the chirp-modulated LITES system. In the LITES system, the focus is typically on the resonant frequency of the QTF. Therefore, we employ the traditional electrical excitation method to measure the frequency response of the QTF near its resonant frequency. Fig. 2(a) shows the frequency calibration curve of the commercial QTF at standard atmospheric pressure using the traditional electric excitation method. The frequency response curve can be well fitted by the Lorentz profile with a correlation coefficient of 0.999. According to the fitting result, the resonant frequency and bandwidth of the QTF are 32756.93 Hz and 3.87 Hz respectively, which leads to a quality factor of 8464. Pure N_2_ is continuously injected into the multi-pass cell with a flow rate of 100 mL/min. The 1 *f* signals are measured individually at different frequency ranges of chirped signals. Fig. 2(b) shows 1 *f* signals at different frequency ranges of 6.0, 21.0, 36.0, 51.0, 66.0 and 81.0 Hz. Fig. 3(a) depicts the fitting result of the 1 *f* signal based on Lorentz profile at the frequency range of 6.0 Hz, and the correlation coefficient is 0.997. The 1 *f* signal does not strictly conform to the Lorentz profile, which may be attributed to the nonlinear intensity response of the tunable diode laser. Based on the peak position of the 1 *f* signal, the QTF resonant frequency is calculated to be 32756.95 Hz. The QTF resonant frequency measured using the traditional electric excitation method is 32756.93 Hz. The measurement discrepancy of the QTF resonant frequency with the two methods is only 0.02 Hz.

Fig. 3(b) records characteristics of 1 *f* signals at different frequency ranges. It can be observed that when the frequency ranges are 6.0, 21.0, 36.0, 51.0, 66.0 and 81.0 Hz, the peak values of the 1 *f* signals are 3.64, 3.56, 3.35, 3.12, 2.91 and 2.72 V, and the minimum values are 2.112, 0.751, 0.439, 0.311, 0.240 and 0.196 V, respectively. The fitting curves yield bias values of 0.961, 0.514, 0.361, 0.282, 0.229 and 0.194 V, respectively, which also represent the theoretical minimum values for the 1 *f* signals. When the frequency range reaches 36.0 Hz, the minimum value of the 1 *f* signal differs from the theoretical minimum by only 0.078 V, corresponding to only 2.3 % of the 1 *f* signal's maximum value. Hence, it can be observed that by extending the frequency range to 36.0 Hz, the profile of a 1 *f* signal can be displayed relatively completely. When the frequency range is increased to 81.0 Hz, the difference between the minimum value of the 1 *f* signal and the theoretical minimum value decreases to 0.002 V. However, the amplitude of the 1 *f* signal decreases by 18.8 %. Therefore, considering both the integrity and amplitude of the 1 *f* signal, the frequency range of 36.0 Hz is selected to better demonstrate the mechanism of the chirp-modulated LITES system.

### 4.2 1 *f* signals at different chirp cycles

**Fig. 4:**
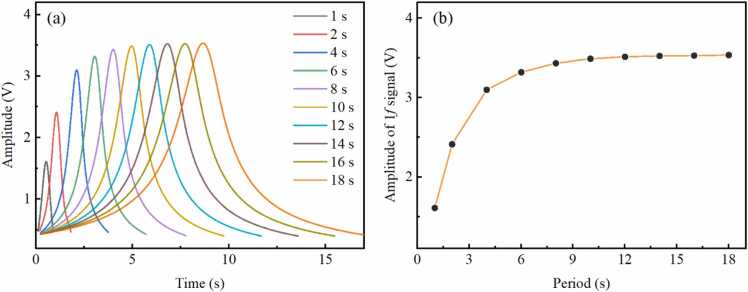
(a) 1 *f* signals and (b) amplitudes of 1 *f* signals measured at different periods of chirped signals.

To investigate the correspondence between 1 *f* signals and chirp cycles, measurements are performed for 1 *f* signals at different cycles of 1 s, 2 s, 4 s, 6 s, 8 s, 10 s, 12 s, 14 s, 16 s, and 18 s. The chirp cycle refers to the total duration of a single chirped signal and the chirped signal is periodically repeated. Pure N_2_ is continuously injected into the multi-pass cell with a flow rate of 100 mL/min. Fig. 4(a) and Fig. 4(b) present 1 *f* signals and amplitudes of 1 *f* signals measured at different chirp cycles, respectively. As the chirp cycle gradually increases, the amplitude of the 1 *f* signal progressively increases. When the chirp cycle exceeds 6 s, the increase in the amplitude of the 1 *f* signal becomes much slower. The 1 *f* signal amplitude increases with longer chirp cycles and then saturates. This is primarily caused by the energy accumulation effect of the QTF. Hence, the chirp cycle of 6 s is selected for subsequent experiments, thereby achieving a relatively fast measurement rate.

### 4.3 1 *f* signals at different modulation amplitudes

**Fig. 5:**
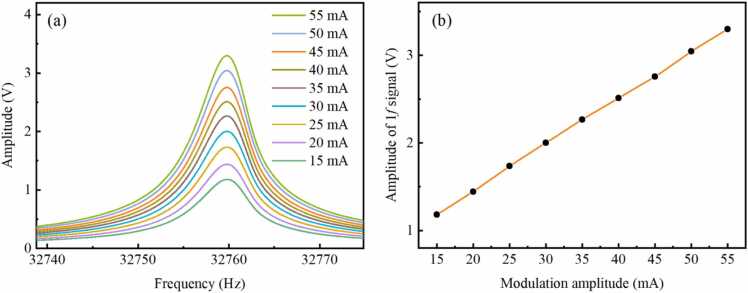
(a) 1 *f* signals and (b) amplitudes of 1 *f* signals measured at different modulation amplitudes.

To investigate the correspondence between 1 *f* signals and amplitudes of chirped signals, measurements are performed for 1 *f* signals at different modulation amplitudes of 15 mA, 20 mA, 25 mA, 30 mA, 35 mA, 40 mA, 45 mA, 50 mA and 55 mA. Pure N_2_ is continuously injected into the multi-pass cell with a flow rate of 100 mL/min. Fig. 5(a) and Fig. 5(b) present 1 *f* signals and amplitudes of 1 *f* signals measured at different modulation amplitudes, respectively. As the modulation current gradually increases, the range of the laser's output light intensity progressively expands. The amplitude of the 1 *f* signal is directly proportional to the light intensity. Hence, it can be observed that the amplitude of the 1 *f* signal proportionally increases with modulation amplitude. According to Eq. (1), a modulation amplitude of 55 mA corresponds to a peak-to-peak modulation current of 110 mA for the laser, with an actual modulation current range spanning from 40 to 150 mA. Limited by the maximum injection current of the laser, the maximum modulation amplitude employed in this experiment is 55 mA, which is adopted for subsequent experiments. However, for LITES sensors equipped with lasers at other spectral bands, it is necessary to constrain the amplitude of the modulation current to prevent it from overlapping with absorption lines of interfering gases.

### 4.4 Concentration calibration

**Fig. 6:**
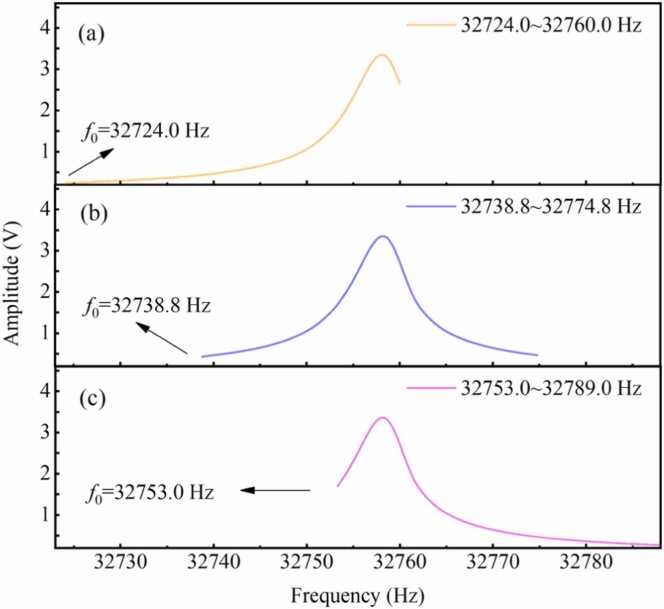
1 *f* signals at chirped frequencies ranging from (a) 32723.0–32759.0 Hz, (b) 32737.8–32773.8 Hz and (c) 32752.0–32788.0 Hz.

In the chirp-modulated LITES system, the measured 1 *f* signal exhibits good agreement with the Lorentz profile. The frequency domain of the Lorentz function is infinite. Even when further expanding the frequency range, the baseline unrelated to absorption can hardly be detected in the spectrum. Consequently, it is challenging to achieve a sweep range that fully covers the 1 *f* response for all concentrations. According to the formula for the Lorentz function, the integral area remains constant within a specific frequency range. Fig. 6(a), Fig. 6(b) and Fig. 6(c) show 1 *f* signals at initial frequencies of 32724.0, 32738.8 and 32753.0 Hz respectively, and the frequency ranges are both 36.0 Hz. When the QTF resonant frequency drifts, real-time correction can be achieved by adjusting the initial frequency of the chirped signal. Through this approach, the resonant frequency of the QTF can be maintained at a specific position within the frequency range of the chirped signal. Therefore, this method ensures the stability of the integral area of the 1 *f* signal within a specific frequency range.

**Fig. 7:**
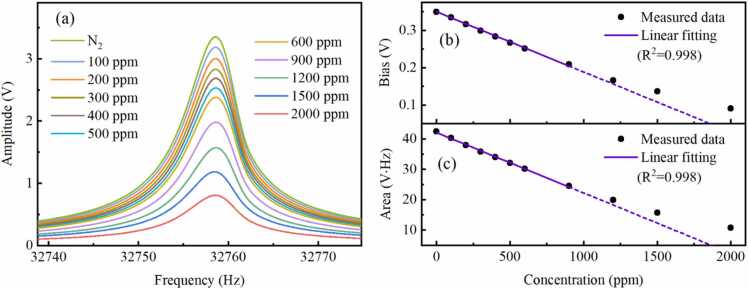
(a) 1 *f* signals, (b) bias and (c) area of 1 *f* signals measured at different gas concentrations with the chirp cycle of 6 s.

**Fig. 8:**
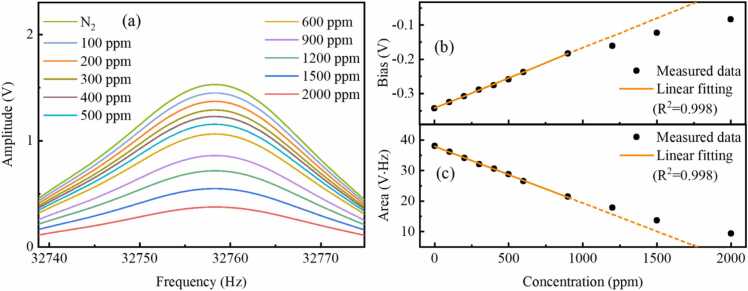
(a) 1 *f* signals, (b) bias and (c) area of 1 *f* signals measured at different gas concentrations with the chirp cycle of 1 s.

Concentration calibration is performed for the chirp-modulated LITES system with the chirp cycle of 6 s. Pure N_2_ and standard CH_4_ samples of 100 ppm, 200 ppm, 300 ppm, 400 ppm, 500 ppm, 600 ppm, 900 ppm, 1200 ppm, 1500 ppm and 2000 ppm are injected into the multi-pass cell successively with a flow rate of 100 mL/min. The 1 *f* signals and corresponding areas at different gas concentrations are recorded in Fig. 7(a) and Fig. 7(c), respectively. Fig. 7(b) shows the relationship between the theoretical 1 *f* signal bias and gas concentration based on Lorentz fitting. While the gas concentration is below 900 ppm, an excellent linear relationship between gas concentration and area of the 1 *f* signal is observed and the correlation coefficient is 0.998. The nonlinearity of the response emerges with gas concentration reaching 1200 ppm. The trend of bias variation with concentration conforms to Eq. (3). The nonlinear effect is primarily determined by the gas absorption law. When the concentration reaches 1200 ppm and the absorbance is 0.12, the gas absorption coefficient will exhibit nonlinear variation. Hence, a nonlinear fitting curve can be adopted to achieve precise measurements of high-concentration gases. For comparison, the concentration calibration curve is also measured at the chirp cycle of 1 s. As shown in Fig. 8(a) and Fig. 8(c), the amplitudes of the 1 *f* signal with this chirp cycle are lower than those measured with the chirp cycle of 6 s. While the gas concentration is below 900 ppm, an excellent linear relationship is observed and the correlation coefficient is 0.998. The nonlinearity of the response emerges with gas concentration reaching 1200 ppm. As shown in Figure8(b), when the chirp cycle is reduced to 1 s, the bias of the Lorentz fit curve for the 1 *f* signal becomes negative, which is due to the demodulation parameters. The trend of bias variation with concentration also conforms to Eq. (3). The measurement results of gas concentration are essentially consistent with the measurement trends observed when the chirp cycle is 6 s. Based on the above analysis, it is reliable to employ the area of the 1 *f* signal for concentration calculations. Furthermore, the bias of the 1 *f* signal does not affect the reliability of concentration calculations.

### 4.5 Allan variance analysis

**Fig. 9:**
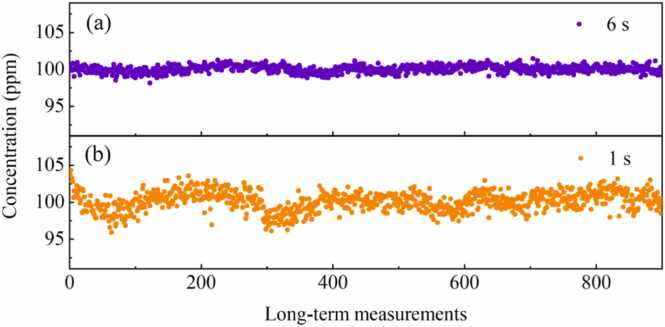
Long-term measurements of chirp-modulated LITES system for the chirp cycle of (a) 6 s and (b) 1 s.

**Fig. 10:**
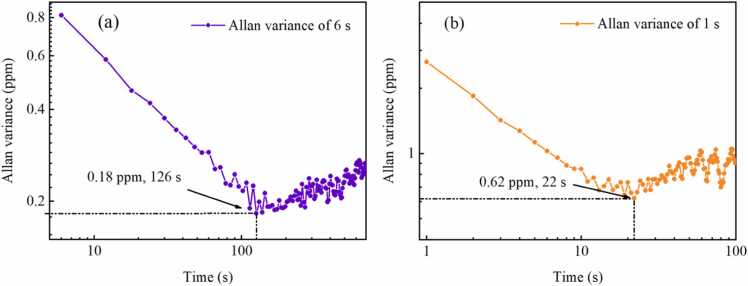
Allan variance of chirp-modulated LITES system for the chirp cycle of (a) 6 s and (b) 1 s.

The 1 *f* signal exhibits a background that correlates with the light intensity. Furthermore, the profile of this 1 *f* signal aligns well with a Lorentz profile. Even when the frequency range is further increased, the baseline unrelated to gas absorption can hardly be identified. Currently, Allan variance is widely applied in LITES systems and can be used to analyze the detection sensitivity of LITES systems under different integration times [53], [54]. Standard CH_4_ sample of 100 ppm is injected into the multi-pass cell continuously with a flow rate of 100 mL/min. Gas concentrations are continuously measured by the chirp-modulated LITES system with the chirp cycle of 6 s, and Allan variance analysis is employed to process the recorded data. Fig. 9(a) displays long-term measurements of chirp-modulated LITES system for the chirp cycle of 6 s. The measurement time for a single concentration is 6 s. The average output optical power of the laser is 7.1 mW, and the time constant of the lock-in amplifier is set to 100 ms. As depicted in Fig. 10(a), the detection limit is 0.81 ppm with an integration time of 6 s. While the integration time is increased to 126 s, the detection limit of the chirp-modulated LITES system reaches a minimum of 0.18 ppm and the corresponding normalized noise equivalent absorption coefficient (NNEA) is 0.37 × 10^−9^ cm^−1^W/Hz^1/2^. Therefore, the chirp-modulated LITES system has the potential to perform high-precision gas measurements.

Fig. 9(b) displays long-term measurements of chirp-modulated LITES system for the chirp cycle of 1 s. The measurement time for a single concentration is 1 s. Compared to the long-term concentration measurements for the chirp cycle of 6 s, the measurement fluctuations are greater. Fig. 10(b) presents the Allan variance analysis for the chirp cycle of 1 s. The detection limit of the chirp-modulated LITES system is 2.67 ppm with an integration time of 1 s. While the integration time is increased to 22 s, the detection limit of the chirp-modulated LITES system reaches a minimum of 0.62 ppm and the corresponding normalized noise equivalent absorption coefficient (NNEA) is 1.27 × 10^−9^ cm^−1^W/Hz^1/2^. For the two chirp cycles, the average number of data points corresponding to the optimal integration time is relatively close. When the chirp cycle falls below the optimal cycle, the system's detection sensitivity decreases accordingly. Therefore, in situations where measurement speed is critical, sacrificing a certain degree of measurement accuracy can enable rapid measurement. Moreover, to accommodate diverse practical application scenarios, we can flexibly select parameters such as the frequency range and chirp cycle of the LITES system, rather than being limited to choosing only the optimal parameters.

### 4.6 Comparison with established detection methods

Table 1 lists the technical parameters of the traditional LITES system based on wavelength modulation technology [19], [55], the beat-frequency LITES system [49], [56] and the chirp-modulation LITES system. It can be seen that the linearity of the three measurement methods is relatively similar. The NNEA of the chirp-modulated LITES system is close to that of the beat-frequency LITES system and is superior to the traditional LITES system. The response time of the chirp-modulated LITES system is superior to that of the traditional LITES system, slightly lower than that of the beat-frequency LITES system. The measurement accuracy of the resonant frequency of the chirp-modulated LITES system can reach up to 0.01 Hz. Moreover, the detection sensitivity of the chirp-modulated LITES system can be enhanced by employing an optical resonant cavity and QTF coated with high-absorption films. Therefore, the chirp-modulated LITES system holds broad application prospects in high-precision measurement applications.

**Table 1:** Technical parameters of the three LITES measurement methods.

Method	Linearity	NNEA (cm^−1^W/Hz^1/2^)	Response time (s)	measurement accuracy of resonant frequency (Hz)
Traditional LITES	0.999	2.33 × 10^−9^	40	\
Beat-frequency LITES	0.999	0.53 × 10^−9^	2	0.2
Chirp-modulation LITES	0.998	0.37 × 10^−9^	6	0.01
	0.998	1.27 × 10^−9^	1	0.01

## 5 Conclusion

Simultaneous precise measurement of QTF resonant frequency and gas concentration is realized in this paper with the chirp-modulated LITES system. Theoretical calculations are performed primarily for the 1 *f* signal of the chirp-modulated LITES system, indicating the simultaneous measurement approach for QTF resonant frequency and gas concentration. The 1 *f* signals are measured individually at different frequency ranges of chirped signals. By extending the frequency range to 36.0 Hz, the complete profile of a 1 *f* signal can be displayed relatively completely, and this frequency range is selected to better demonstrate the mechanism of the chirp-modulated LITES system. Based on the peak position of the 1 *f* signal, the QTF resonant frequency is calculated to be 32756.95 Hz. Compared with the traditional electric excitation method, the measurement deviation is only 0.02 Hz. Moreover, real-time correction can be achieved by adjusting the initial frequency of the chirped signal while the QTF resonant frequency drifts. Variation trends of the 1 *f* signal under different modulation periods and currents are also investigated. A modulation period of 6 s and a modulation amplitude of 55 mA are employed to achieve a relatively fast measurement rate and a higher 1 *f* signal amplitude. While the gas concentration is below 900 ppm, the chirp-modulated LITES system shows an excellent linear response and the correlation coefficient is 0.998. To achieve precise measurements of high-concentration gases, a nonlinear fitting curve can be adopted. According to Allan variance analysis, the detection limit of the chirp-modulated LITES system reaches a minimum of 0.18 ppm with an integration time of 126 s. Moreover, the characteristics of the chip-modulated LITES system with the chirp cycle of 1 s are also investigated. In situations where measurement speed is critical, sacrificing a certain degree of measurement accuracy can enable rapid measurement. The chirp-modulated LITES system offers a novel approach for simultaneously and precisely measuring the QTF resonant frequency and gas concentration. Based on the 1 *f* signal, the resonant frequency of the QTF can be measured in real time. If the measured resonant frequency of the QTF decreases or increases by 1.0 Hz at any given moment, the initial frequency of the chirped signal is adjusted accordingly by decreasing or increasing by 1.0 Hz. This adjustment is performed continuously in real time. Through this approach, the resonant frequency of the QTF can be maintained at a specific position within the frequency range of the chirped signal, enabling real-time calibration of the frequency drift. This technique eliminates the impact of resonant frequency drift on spectral measurement results, effectively enhancing the stability of the LITES system in long-term measurement applications.

## CRediT authorship contribution statement

**Lu Yao:** Writing – review & editing, Conceptualization. **Xinyao Chen:** Resources, Methodology, Investigation. **Xiang Chen:** Writing – original draft, Validation, Methodology, Data curation. **Ruifeng Kan:** Supervision, Resources, Methodology. **Hao Liu:** Writing – review & editing, Methodology, Conceptualization. **Mai Hu:** Methodology, Investigation.

## Declaration of Competing Interest

The authors declare that they have no known competing financial interests or personal relationships that could have appeared to influence the work reported in this paper.

## Data Availability

Data will be made available on request.
